# Zinc Alleviates Diabetic Muscle Atrophy via Modulation of the SIRT1/FoxO1 Autophagy Pathway Through GPR39

**DOI:** 10.1002/jcsm.13771

**Published:** 2025-03-03

**Authors:** Xing Yu, Xiaojun Chen, Weibin Wu, Huibin Tang, Yunyun Su, Guili Lian, Yujie Zhang, Liangdi Xie

**Affiliations:** ^1^ Department of Geriatrics The First Affiliated Hospital of Fujian Medical University Fuzhou Fujian China; ^2^ Fujian Hypertension Research Institute The First Affiliated Hospital of Fujian Medical University Fuzhou Fujian China; ^3^ Clinical Research Center for Geriatric Hypertension Disease of Fujian Province The First Affiliated Hospital of Fujian Medical University Fuzhou Fujian China; ^4^ Branch of National Clinical Research Center for Aging and Medicine The First Affiliated Hospital of Fujian Medical University Fuzhou Fujian China; ^5^ Department of Geriatrics National Regional Medical Center Binhai Campus of the First Affiliated Hospital Fujian Medical University Fuzhou Fujian China

**Keywords:** autophagy, GPR39, muscle atrophy, SIRT1/FoxO1, zinc

## Abstract

**Background:**

Muscle atrophy is a severe complication of diabetes, with autophagy playing a critical role in its progression. Zinc has been shown to alleviate hyperglycaemia and several diabetes‐related complications, but its direct role in mediating diabetic muscle atrophy remains unclear. This study explores the potential role of zinc in the pathogenesis of diabetic muscle atrophy.

**Methods:**

In vivo, C57BL/6J mice were induced with diabetes by streptozotocin (STZ) and treated with ZnSO₄ (25 mg/kg/day) for six weeks. Gastrocnemius muscles were collected for histological analysis, including transmission electron microscopy (TEM). Serum zinc levels were measured by ICP‐MS. Protein expression was evaluated using immunofluorescence (IF), immunohistochemistry (IHC) and Western blotting (WB). Bioinformatics analysis was used to identify key genes associated with muscle atrophy. In vitro, a high‐glucose‐induced diabetic C2C12 cell model was established and received ZnSO₄, rapamycin, SRT1720, TC‐G‐1008, or GPR39‐CRISPR Cas9 intervention. Autophagy was observed by TEM, and protein expression was assessed by IF and WB. Intracellular zinc concentrations were measured using fluorescence resonance energy transfer (FRET).

**Results:**

In vivo, muscle atrophy, autophagy activation, and upregulation of SIRT1 and FoxO1, along with downregulation of GPR39, were confirmed in the T1D group. ZnSO₄ protected against muscle atrophy and inhibited autophagy (T1D + ZnSO₄ vs. T1D, all *p* < 0.0001), as evidenced by increased grip strength (212.40 ± 11.08 vs. 163.90 ± 10.95 gf), gastrocnemius muscle index (10.67 ± 0.44 vs. 8.80 ± 0.72 mg/g), muscle fibre cross‐sectional area (978.20 ± 144.00 vs. 580.20 ± 103.30 μm^2^), and serum zinc levels (0.2335 ± 0.0227 vs. 0.1561 ± 0.0123 mg/L). ZnSO₄ down‐regulated the expression of Atrogin‐1 and MuRF1, and decreased the formation of autophagosomes in the gastrocnemius muscle of T1D mice (all *p* < 0.0001). RNA‐seq analysis indicated activation of the SIRT1/FoxO1 signalling pathway in diabetic mice. ZnSO₄ down‐regulated LC3B, SIRT1 and FoxO1, while upregulating P62 and GPR39 (all *p* < 0.05). In vitro, muscle atrophy, autophagy activation, and down‐regulation of GPR39 were confirmed in the diabetic cell model (all *p* < 0.05). Both ZnSO₄ and TC‐G‐1008 down‐regulated Atrogin‐1, LC3B, SIRT1, and FoxO1, and up‐regulated P62 and GPR39, inhibiting autophagy and improving muscle atrophy (all *p* < 0.05). The beneficial anti‐atrophic effects of ZnSO₄ are diminished following treatment with SRT1720 or RAPA. Upon GPR39 knockout, SIRT1, FoxO1, and Atrogin‐1 were upregulated, while P62 was downregulated. Intracellular zinc concentrations in ZnSO₄‐treated group remained unchanged (*p* > 0.05), indicating that zinc supplementation did not affect zinc ion entry but acted through the cell surface receptor GPR39.

**Conclusion:**

ZnSO_4_ inhibits excessive autophagy in skeletal muscle and alleviates muscle atrophy in diabetic mice via the GPR39‐SIRT1/FoxO1 axis. These findings suggest that zinc supplementation may offer a potential therapeutic strategy for managing diabetic muscle atrophy.

## 1 Introduction

Diabetes mellitus constitutes one of the most significant public health challenges, characterized by a rapid increase in prevalence globally in recent years, resulting in adverse health outcomes and substantial medical expenses [1]. A notable complication of diabetes is skeletal muscle dysfunction, which is particularly pronounced in patients with Type 1 diabetes (T1D) [2, 3]. Monaco et al. [4] reported that T1D accelerates muscle aging, with a 20.0% incidence of muscle loss in patients aged 65 and older, significantly higher than the 8.1% observed in individuals with Type 2 diabetes (T2D). Moreover, muscle atrophy adversely impacts the physical and metabolic health of patients with T1D, increasing mortality risk [5]. Despite the critical importance of addressing skeletal muscle atrophy, preventive and therapeutic strategies for this condition remain largely in their infancy.

Zinc is recognized as an essential micronutrient integral to health [6]. Studies have demonstrated that individuals with sarcopenia exhibit significantly lower serum zinc levels compared to those without the condition [7]. Further research has elucidated the multifaceted protective effects of zinc supplementation, which is increasingly employed in the prevention and management of diabetes [8]. However, the specific role of zinc in the context of diabetic muscle atrophy has yet to be thoroughly investigated. Recently, it was reported that extracellular Zn^2+^ bind to various cell surface proteins, with G protein‐coupled receptor 39 (GPR39) identified as the sole specific target for Zn^2+^, demonstrating a high degree of selectivity [9, 10]. Silencing GPR39 has been shown to markedly diminish the inhibitory effects of zinc supplementation on calcification and apoptosis in human valve interstitial cells in vitro [11]. Our previous research [12] indicated that zinc regulates TNF‐alpha‐induced Protein 3 (A20/TNFAIP3) expression via the GPR39 receptor, providing protective effects against monocrotaline‐induced pulmonary hypertension in rats. Based on these previous research work, we hypothesize that zinc ions are normally present in skeletal muscle under physiological conditions, while their levels are diminished in individuals with diabetes. Supplementation of exogenous Zn^2+^ is proposed to activate GPR39, triggering a cascade of responses that alleviate muscle atrophy in diabetic mice.

Autophagy, a lysosome‐dependent self‐degradation mechanism, is crucial for the degradation and recycling of dysfunctional cellular components [13, 14]. Research has highlighted the central role of autophagy in the degradation of muscle proteins induced by diabetes [15]. This process can activate significant protein breakdown, utilizing amino acids derived from the citric acid cycle as fuel for adenosine triphosphate production, thereby sustaining energy balance. In insulin‐deficient T1D mice, autophagy is activated to compensate for energy deficits, contributing to muscle atrophy. Silent information regulator 1 (SIRT1), an NAD^+^‐dependent class III histone deacetylase, regulates various biological processes, including inflammatory responses, oxidative stress, apoptosis, and autophagy [16]. Forkhead box‐O1 (FoxO1), a member of the forkhead transcription factor family, serves as a crucial deacetylation substrate for SIRT1 [17]. Nevertheless, the role of SIRT1/FoxO1‐mediated autophagy in diabetic muscle atrophy remains largely unexamined.

In this study, we first established a diabetic mouse model to assess the protective effects of zinc supplementation against muscle atrophy. Subsequent transcriptomic analysis revealed up‐regulation of autophagy‐related genes in the gastrocnemius muscles of diabetic mice. Building on these findings, we conducted in vitro studies using C2C12 cells to further explore the role of zinc and zinc transporters in muscle atrophy, as well as the underlying mechanisms by which zinc modulates this process.

## 2 Material and Methods

### 2.1 Animal Model and Treatment

All experimental procedures were conducted in accordance with national health guidelines and received approval from the Animal Welfare and Ethics Committee of Fujian Medical University (approval no. FJMU IACUC 2021‐0378, Fuzhou, China). Male C57BL/6J mice, aged 6–7 weeks, were sourced from Shanghai SLACCAS Laboratory Animal Co., Ltd. Mice were housed at 23 ± 1°C with a 12‐h light/dark cycle and given ad libitum access to water and a standard diet.

Diabetes was induced via a single intraperitoneal injection of streptozotocin (STZ, 150 mg/kg, Sigma‐Aldrich, USA) dissolved in 0.1 M citrate buffer (pH 4.5) [18]. Control mice (Ctrl) received an equivalent volume of sodium citrate buffer. Blood glucose levels were measured 72 h post‐injection using a glucose meter (Roche, Switzerland), and mice with levels above 300 mg/dL (16.7 mmol/L) were included. Diabetic mice were randomly divided into T1D (saline‐treated) or T1D + ZnSO₄ (25 mg/kg zinc sulphate‐treated) groups. Throughout the experiment, daily monitoring of food intake and water consumption occurred, while body weight, grip strength, and fasting blood glucose levels were recorded weekly.

After six weeks of treatment, mice were euthanized, and blood and skeletal muscle samples were collected. Absolute gastrocnemius muscle mass was determined as the average weight of the left and right muscles. The gastrocnemius muscle index (GMI), calculated as absolute muscle mass (mg) divided by body weight (g), was used to normalize variations in body weight, ensuring a more objective comparison of treatment effects on muscle mass.

### 2.2 Grip Strength Test

Grip strength measurements were conducted as described previously [19] using the YLS13A grip strength meter (Nuoleixinda, Tianjin, China). Each mouse was tested six times, and the average was recorded as absolute limb muscle strength. To account for body weight differences, relative grip strength was calculated by normalizing absolute grip strength to body weight. This method enabled precise comparisons of relative grip strength across groups.

### 2.3 Measurement of Serum Zinc Concentration Using Inductively Coupled Plasma‐Mass Spectrometry (ICP‐MS)

Serum zinc levels in mice were quantified using ICP‐MS (PerkinElmer, USA) [12]. Serum samples were prepared through microwave digestion. Standard solutions at concentrations of 0.05, 0.5, 5, 50, and 500 ppb were prepared to generate a standard curve. All samples were automatically introduced into the system using a fully automated sample loader, allowing for accurate measurement of zinc levels.

### 2.4 Histological Analysis, Transmission Electron Microscope (TEM), Western Blot and Cell Immunofluorescence (IF) Staining

Detailed protocols and antibody information are provided in the supplementary material Methodology sections.

### 2.5 Bioinformatics Analysis

Principal Component Analysis (PCA) was performed using the R package gmodels (https://www.r‐project.org/). Differentially expressed genes (DEGs), Kyoto Encyclopedia of Genes and Genomes (KEGG) pathway analysis, Protein–Protein Interaction (PPI) networks and Gene Set Enrichment Analysis (GSEA) were conducted as previously described [20].

### 2.6 Cell Culture, Differentiation and Treatment

C2C12 myoblasts (catalogue no. #CL‐0044) were purchased from Procell Life Science & Technology Co., Ltd (Wuhan, China) and cultured in DMEM/high glucose (HG) medium (BasalMediaTechnologies Co., Ltd, Shanghai, China), supplemented with 10% foetal bovine serum, 100 IU/mL penicillin and 100 μg/mL streptomycin (Invitrogen, USA). Upon reaching 80%–90% confluence, the medium was replaced with differentiation medium consisting of DMEM supplemented with 2% heat‐inactivated horse serum and antibiotics. Cells were cultured for 5 days, with medium replaced daily. Differentiated myotubes were confirmed through IF staining. To simulate the diabetic myotube environment, glucose concentration in the medium was reduced to 5 mM for at least 18 h post‐differentiation, followed by an increase to 50 mM for a defined duration.

Myotube cells were treated with HG concentrations, with or without the addition of ZnSO_4_ (30 μM, Cat No: 7733‐02‐0, Macklin, China), the autophagy agonist rapamycin (RAPA, Cat No: S1039, Selleck, China; 50 nm), the SIRT1‐specific agonist SIRT1720 (Cat No: S1129, Selleck, China; 1 μm), and the GPR39‐specific agonist TC‐G‐1008 (Cat No: S6759, Selleck, China). These treatments aimed to investigate the regulatory mechanisms of ZnSO_4_ and the SIRT1/FoxO1 signalling pathway. Following treatments, cells were collected for subsequent analyses.

### 2.7 CRISPR/Cas9 Gene Knockdown

CRISPR/Cas9 technology was used to generate GPR39 knockout C2C12 cell lines (GPR39 KO) following established protocols [21]. The LentiGuide‐Puro vector (Plasmid #52963, Addgene) was used, and virus was harvested 48 h post‐transfection. Transfected cells were selected with puromycin (2 μg/mL for 2 days), followed by monoclonal isolation in 96‐well plates. Single clones were validated via Sanger sequencing, and knockout efficiency was confirmed by Western blot and IF. The sgRNA sequences targeting GPR39 were: sgRNA1: ATGGCTGTGATCGATGACAC, sgRNA2: TGATCAGGTACACCAAGATG (5′ → 3′).

### 2.8 Quantification of Zinc Concentration by Fluorescence Resonance Energy Transfer (FRET) Measurement

FRET was performed as previously described [12]. A two‐colour FRET experiment utilized Cyan Fluorescent Protein, with imaging conducted using a 40× objective lens on a 405 nm laser confocal microscope (Zeiss LSM800, Germany) to visualize C2C12 cells. For in‐situ calibration, cells were imaged in a confocal glass dish containing 0.5 mL of Krebs‐Hepes‐Bicarbonate (KHBP) buffer without additives. Subsequently, 0.5 mL of KHB buffer containing 100 μM TPEN (Selleck) was added, followed by KHBP buffer with 200 μM ZnCl2 (Sigma) and 10 mM of the Zn^2+^‐specific ionophore pyrithione (Selleck). The steady‐state fluorescence intensity ratio between the acceptor and donor was measured to establish minimum and maximum values, which were used to calculate free Zn^2+^ concentration with the formula: [Zn^2+^] = Kd × (R_max_ − R) / (R − R_min_). R_max_ was determined by chelating intracellular zinc with 50 μM TPEN, while R_min_ was achieved by saturating the cells with 100 μM ZnCl2 in the presence of 5 μM pyrithione.

### 2.9 Reverse Transcription (RT)‐PCR Amplification and RT‐qPCR

Total RNA was isolated from mouse gastrocnemius muscles using TRIzol (Invitrogen, Carlsbad, CA, USA). RT‐qPCR was conducted using Hieff® qPCR SYBR Green Master Mix (Yeasen Biotech, Shanghai, China) on the LightCycler® 96 System (Roche Diagnostics, Mannheim, Germany) [22]. Amplified products were resolved on a 2% agarose gel in TAE buffer and visualized with 0.5 μg/mL ethidium bromide. Gene expression changes were quantified using the comparative CT (2^−ΔΔCt^) method, normalized to GAPDH as a control. The primer pairs used for amplification are listed in Supplementary Table 1.

### 2.10 Statistical Analysis

Data are presented as mean ± standard deviation (SD). Statistical analysis was performed using SPSS 20.0 software (IBM Corp., Armonk, NY, USA), with results visualized using GraphPad Prism 8 software (GraphPad Software Inc., San Diego, CA, USA). Group differences were evaluated using unpaired Student's *t*‐test or one‐way ANOVA, followed by Tukey's post hoc test in GraphPad Prism 8. Pearson correlation analysis was employed for simple linear correlation analysis. A *p*‐value of < 0.05 was considered statistically significant.

## 3 Results

### 3.1 ZnSO_4_ Alleviates STZ‐Induced Diabetic Muscle Atrophy

**FIGURE 1:**
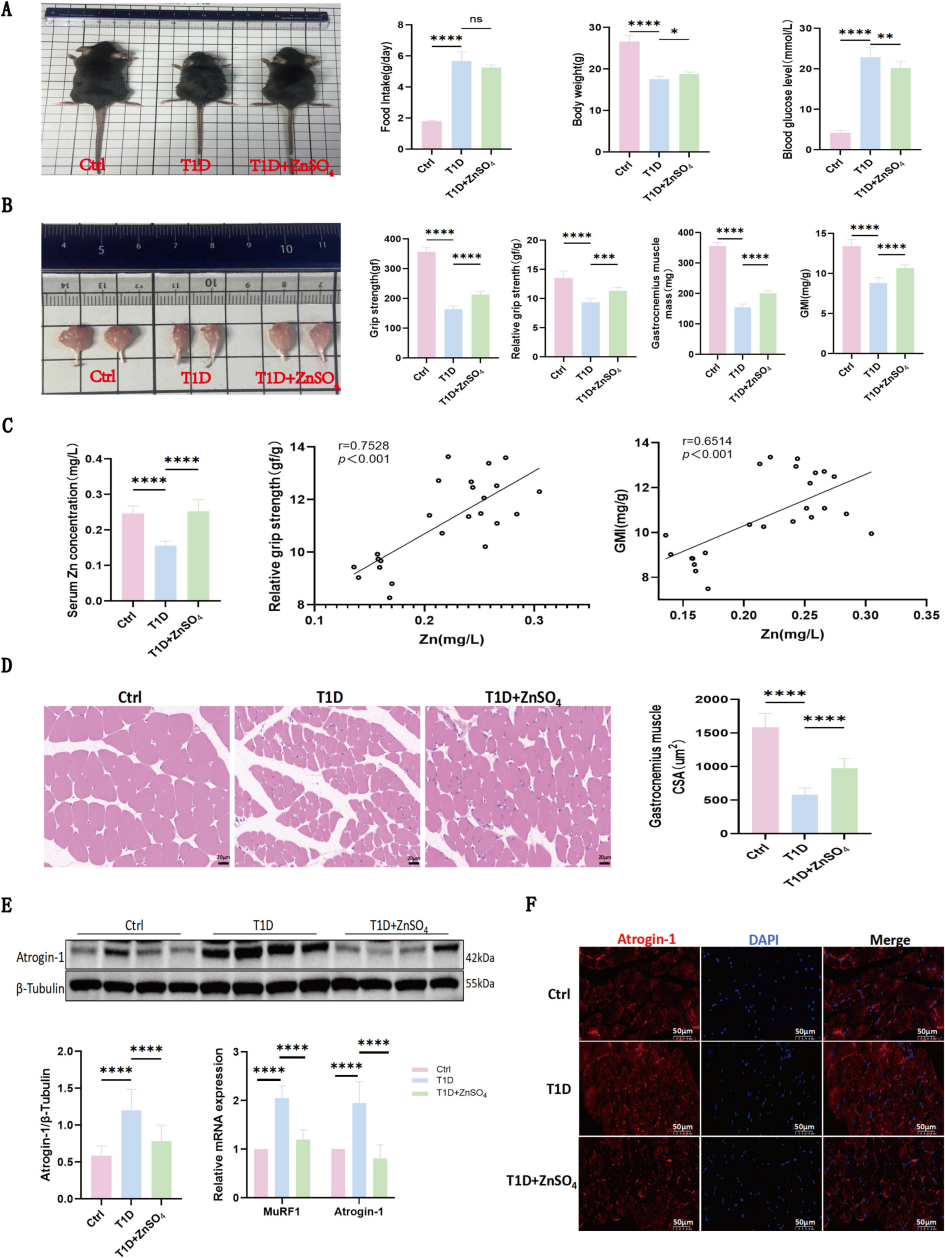
ZnSO₄ alleviates STZ‐induced diabetic muscle atrophy. (A) The general morphology, food intake, body weight, and blood glucose levels of mice in the control, T1D and T1D + ZnSO₄ groups. (B) Macroscopic view of the gastrocnemius muscle (GM); Grip strength, relative grip strength, GM weight and gastrocnemius muscle index (GMI, normalized to body weight) in the three groups. (C) Serum zinc concentrations across different groups and the correlation analysis of serum Zn levels with relative grip strength and GMI. (D) Haematoxylin and Eosin staining of gastrocnemius muscle (scale bar: 20 μm) and statistical analysis of cross‐sectional area (CSA) in the different groups. (E) Representative western blot analysis of Atrogin‐1 expression, quantification of the Atrogin‐1/β‐Tubulin ratio across different groups, and the mRNA expression levels of both Atrogin‐1 and MuRF1 were conducted. (F) Immunofluorescence staining of Atrogin‐1 in muscle tissue. Data are presented as mean ± SD (*n* = 8). *p < 0.05, **p < 0.01, ***p < 0.001, ****p < 0.0001; Abbreviations: CSA: cross‐sectional area; Ctrl: control group; GMI: gastrocnemius muscle index; ns: not significant. T1D: Type 1 diabetes group.

Diabetic mice, characterized by sparse and dull fur and reduced responsiveness, showed significant increases in food intake, elevated blood glucose levels, and notable decreases in body weight compared to control mice with dense and glossy fur; however, following ZnSO_4_ treatment, these T1D mice demonstrated gradual weight gain, reduced blood glucose levels (*p* < 0.05), and a slight, non‐significant decrease in food intake (*p* > 0.05), as illustrated in Figure 1A. At the end of the experiment, visual inspection revealed smaller hindlimb muscles in T1D mice compared to controls, but ZnSO_4_ treatment helped reduce this muscle atrophy, and further analysis indicated that ZnSO_4_ also led to significant improvements in both limb grip strength and relative grip strength, which were reduced in T1D mice, along with increases in gastrocnemius muscle weight and GMI, which were significantly lower in T1D mice compared to controls (Figure 1B). Serum zinc levels were significantly diminished in T1D mice compared to controls, while ZnSO_4_ supplementation restored serum zinc levels to those of the control group. Pearson correlation analysis indicated there was a positive correlation between serum zinc levels and both relative muscle strength and GMI in T1D mice (Figure 1C). Additionally, the average cross‐sectional area of gastrocnemius muscle fibres was significantly reduced in T1D mice; ZnSO_4_ treatment effectively increased this cross‐sectional area (Figure 1D).

The presence and progression of muscle atrophy were associated with significant alterations in muscle atrophy markers, including muscle atrophy F‐Box protein (Atrogin‐1) and muscle‐specific ring finger protein 1 (MuRF1). Notably, Atrogin‐1 protein expression was significantly upregulated in T1D mice, but ZnSO_4_ treatment effectively reversed this trend, leading to downregulation of Atrogin‐1. Consistent changes were observed at the mRNA level for both Atrogin‐1 and MuRF1 (Figure 1E). Immunofluorescence analysis revealed significantly higher fluorescence intensity of Atrogin‐1 in the muscles of the T1D group compared to controls, with a corresponding decrease in the ZnSO_4_ group (Figure 1F). These findings indicate successful establishment of a diabetic muscle atrophy model, with ZnSO_4_ demonstrating efficacy in alleviating STZ‐induced muscle atrophy.

### 3.2 ZnSO_4_ Inhibits Skeletal Muscle Autophagy in Diabetic Mice

**FIGURE 2:**
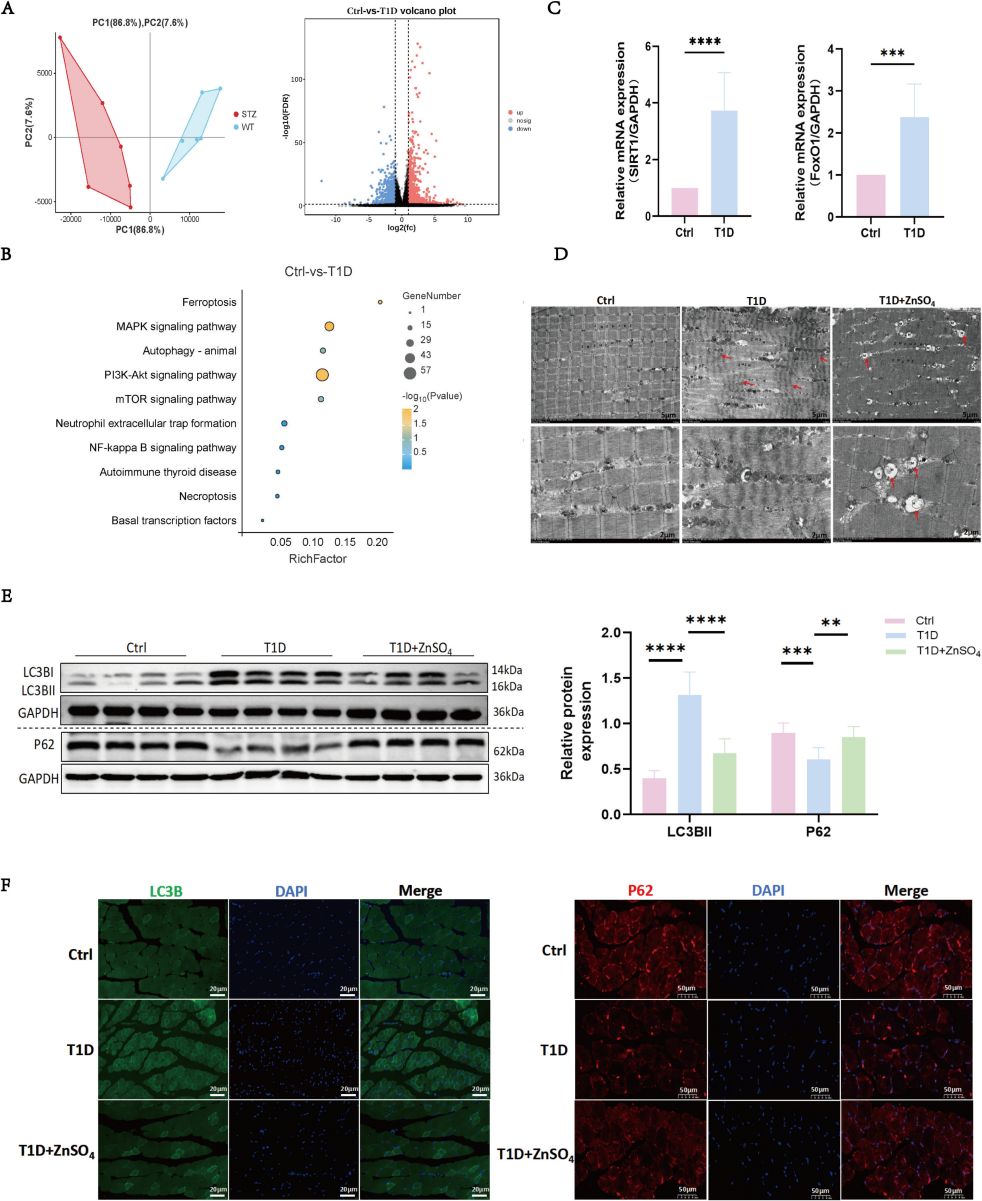
ZnSO₄ inhibits skeletal muscle autophagy in diabetic mice. (A) Principal component analysis was conducted on transcriptome expression in control and T1D gastrocnemius muscles and a volcano plot was generated to visualize differentially expressed genes (DEGs) between T1D and control muscles, where genes with an absolute fold change ≥2 and *p* < 0.05 are highlighted, with the dashed horizontal line indicating *p* = 0.05 and vertical dashed lines showing two‐fold upregulation or downregulation. (B) Kyoto Encyclopedia of Genes and Genomes (KEGG) pathway enrichment analysis of upregulated DEGs in T1D atrophic muscles, showing the top 10 enriched pathways. (C) RT‐qPCR analysis of SIRT1 and FoxO1 expression in GM. (D) Transmission electron microscopy (TEM) images of GM from control, T1D, and T1D + ZnSO₄ mice. Red arrows indicate contraction bands; M: mitochondria; T: transverse tubules; Spr: sarcoplasmic reticulum; Z: Z‐line; H: H‐band; ASS: autophagic lysosome; AP: autophagosome. (E) Representative immunoblots of LC3BII and P62 in GM, with relative expression levels normalized to GAPDH. (F) Immunofluorescence analysis of LC3BII and P62 in GM tissue. Data are presented as mean ± SD (*n* = 8). *p < 0.05, **p < 0.01, ***p < 0.001, ****p < 0.0001. Ctrl: control group; T1D: Type 1 diabetes group.

To investigate the mechanism by which ZnSO_4_ mediates muscle atrophy, RNA sequencing analysis was performed on gastrocnemius muscles from control and T1D mice. Principal component analysis confirmed good reproducibility among replicated samples within both groups (Figure 2A). Differential gene screening identified 1970 DEGs (Figure 2A), with a heatmap displaying upregulated DEGs related to autophagy in T1D muscles (Supplementary Figure 1A). KEGG pathway analysis revealed enrichment of DEGs in autophagy (Figure 2B). The DEGs were analysed using the String database to construct a PPI network, identifying four intersection genes through six algorithms: BCL2L11, SIRT1, FoxO1 and GSK3β(Supplementary Table 2). GSEA of the target pathway indicated activation of autophagy in the STZ group of mice (Supplementary Figure 1B). RT‐qPCR analysis revealed significant increases in the mRNA expression levels of BCL2L11, SIRT1, FoxO1 and GSK3β in the gastrocnemius muscle of T1D mice compared to controls (Figure 2C and Supplementary Figure 1C).

Electron microscopy revealed an abundance of autophagolysosomes and autophagosomes in the intermyofibrillar and subsarcolemmal regions of T1D muscle (Figure 2D). Subsequent IF staining and Western blotting analyses demonstrated increased expression of LC3BII and decreased expression of P62 in the gastrocnemius muscle of T1D mice, indicating enhanced autophagy (Figure 2E,F). In contrast, pretreatment with ZnSO_4_ resulted in downregulation of autophagy‐related molecules. These findings suggest that excessive autophagy in the skeletal muscles of diabetic mice can be inhibited by ZnSO_4_.

### 3.3 ZnSO_4_ Pretreatment Activates GPR39 and Downregulates the SIRT1/FoxO1 Pathway in T1D Mice

**FIGURE 3:**
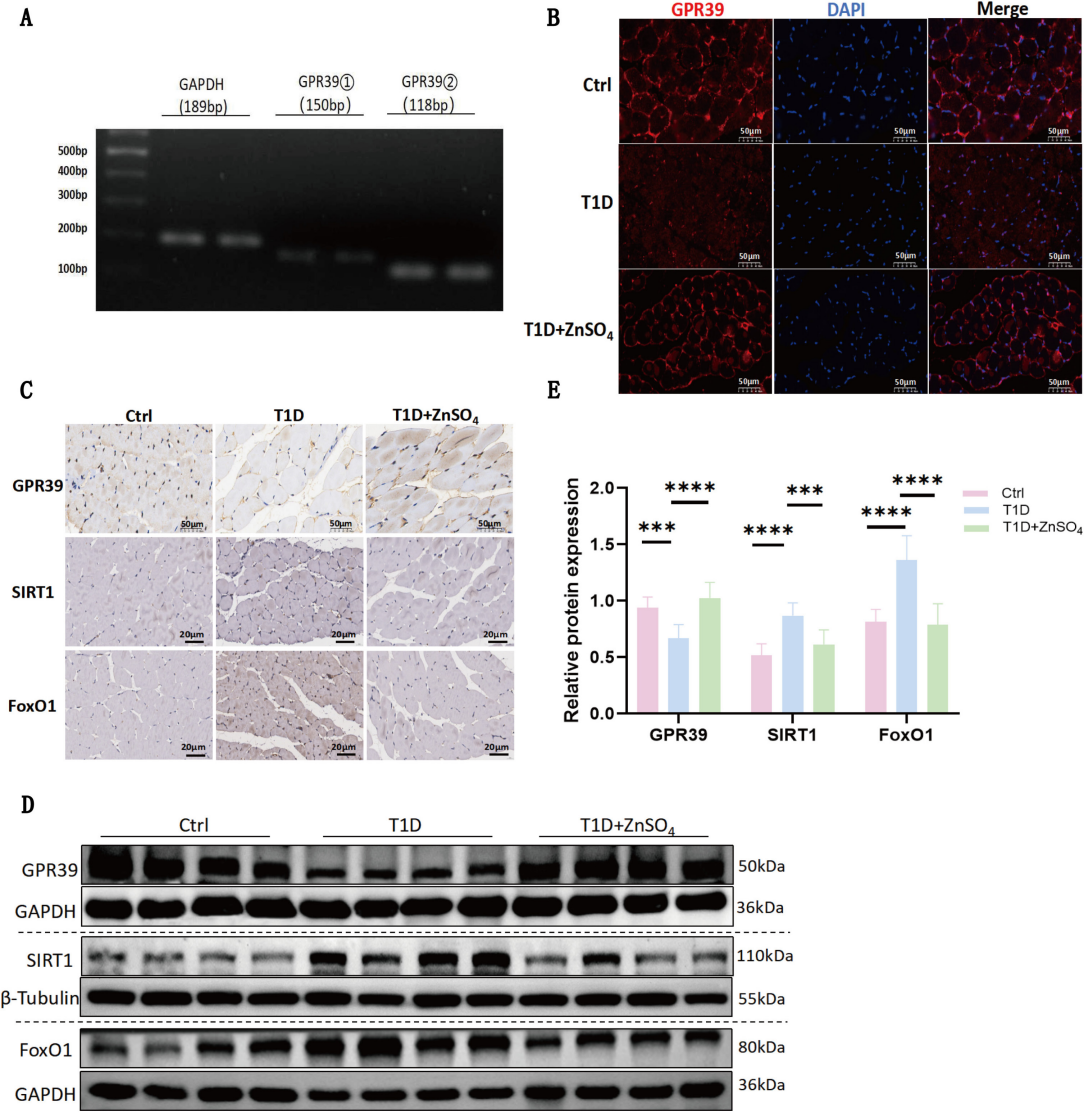
ZnSO₄ activates GPR39 and downregulates the SIRT1/FoxO1 pathway in T1D mice. (A) RT‐PCR analysis of GPR39 expression (using two primer pairs: 189 bp and 150 bp) in gastrocnemius muscle. (B) Immunofluorescence staining of GPR39 in muscle tissue. (C) Immunohistochemical staining of GPR39, SIRT1 and FoxO1 in GM from different groups. (D) Representative western blot analysis of GPR39, SIRT1 and FoxO1 in GM. (E) Relative expression levels of GPR39, SIRT1 and FoxO1 normalized to GAPDH or β‐Tubulin, based on densitometric analysis. Data are presented as mean ± SD (*n* = 8). *p < 0.05, **p < 0.01, ***p < 0.001, ****p < 0.0001.

Agarose gel electrophoresis results confirmed abundant expression of GPR39 in gastrocnemius muscle tissue (Figure 3A). IHC and IF staining demonstrated specific localization of GPR39 in the gastrocnemius muscle (Figure 3B,C). Expression levels of GPR39 were significantly reduced in the T1D group; however, this expression was restored following ZnSO_4_ intervention. Western blot analysis further corroborated the decrease in GPR39 expression in T1D mice, with subsequent upregulation observed post‐treatment (Figure 3B–E).

Additionally, the study investigated the interplay between SIRT1, FoxO1 and the autophagy process in diabetic muscle atrophy. Western blot analysis indicated that expression levels of SIRT1 and FoxO1 were downregulated in the gastrocnemius muscle of T1D mice following ZnSO_4_ treatment (Figure 3D,E). Immunohistochemical analysis revealed increased expression of SIRT1 and FoxO1 in T1D muscles, with restoration of these proteins following ZnSO4 treatment (Figure 3C). Collectively, these findings suggest that ZnSO_4_ pretreatment activates GPR39, downregulates the SIRT1/FoxO1 pathway in T1D mice, and inhibits autophagy‐related factors.

### 3.4 ZnSO_4_ Attenuates Autophagy in C2C12 Myotubes and Alleviates HG‐Induced Myotube Atrophy

**FIGURE 4:**
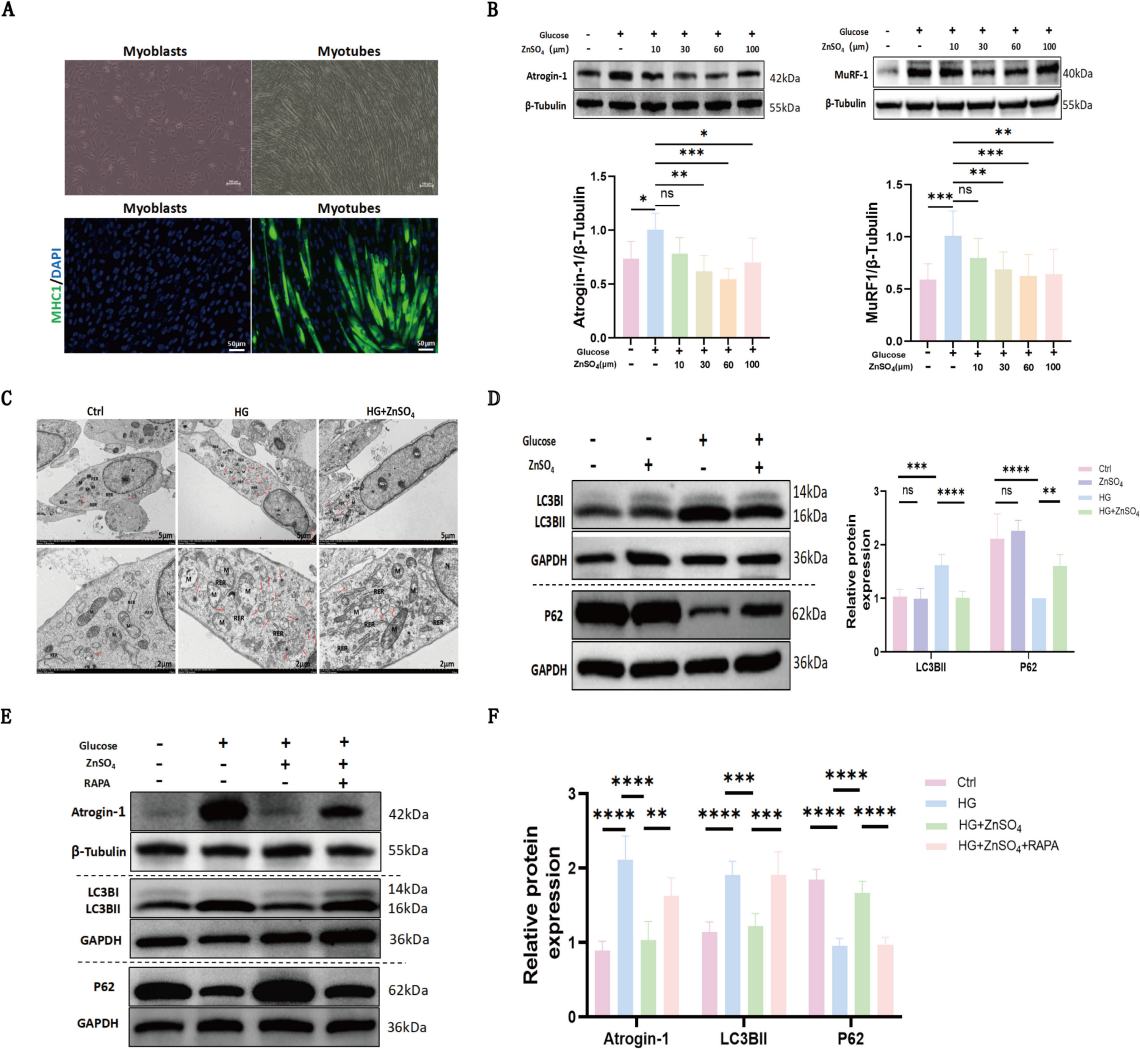
ZnSO₄ inhibits autophagy in C2C12 myotubes and alleviates high glucose‐induced myotube atrophy. (A)The morphological characteristics of C2C12 myoblasts and myotubes (scale bar: 100 μm) are accompanied by immunofluorescence staining of myosin heavy chain 1 (MHC1, green) in these cells, along with DAPI nuclear counterstaining (scale bar: 50 μm).(B) Western blot analysis of Atrogin‐1 and MuRF1 expression in C2C12 myotubes treated with 50 mM glucose ± ZnSO₄ (10, 30, 60, 100 μM) for 72 h. (C) TEM micrographs of myotubes treated with 50 mM glucose ± 30 μM ZnSO₄ for 72 h. Red arrows indicate autophagolysosomes; N: nucleus; NU: nucleolus; M: mitochondria; RER: rough endoplasmic reticulum. (D) Western blot analysis of LC3BII, P62 and GAPDH expression in C2C12 myotubes treated with or without ZnSO₄ (30 μM) and glucose (50 mM) for 72 h. (E–F) Western blot analysis of Atrogin‐1, LC3BII, P62, GAPDH and β‐Tubulin in myotubes treated with glucose (50 mM), ZnSO₄ (30 μM) and RAPA (50 nM) for 72 h. Data are presented as mean ± SD (*n* = 5). *p < 0.05, **p < 0.01, ***p < 0.001, ****p < 0.0001; ns: not significant.

To characterize the direct effects of ZnSO_4_ on muscle cells, an in vitro C2C12 muscle cell model was employed. C2C12 myoblasts displayed a mononuclear spindle‐shaped morphology, while fully differentiated myotubes exhibited a multinucleated slender structure (Figure 4A). Differentiation was confirmed via immunofluorescence using a monoclonal antibody against myosin heavy chain 1 (MHC1), a specific biomarker for myotubes (Figure 4A). In C2C12 myotubes subjected to high glucose (HG; 50 mM), elevated expression of muscle atrophy markers, specifically Atrogin‐1 and MuRF1, was observed. C2C12 myotubes were treated with 10, 30, 60, and 100 μM ZnSO_4_ for 72 h, both in the presence and absence of HG. ZnSO_4_ treatment effectively mitigated the increase in Atrogin‐1 and MuRF1 expression induced by HG (Figure 4B). For subsequent experiments, 30 μM ZnSO_4_ was utilized alongside a 72‐h incubation.

Transmission electron microscopy revealed membrane disruption and nuclear degeneration in HG‐treated myotubes, along with significant mitochondrial swelling, loss of mitochondrial cristae, vacuolar structures and numerous autophagic lysosomes (Figure 4C). In contrast, mild organelle and mitochondrial swelling with reduced autophagy was observed in the ZnSO_4_ treatment group. Furthermore, HG treatment led to increased LC3B‐II protein expression and decreased P62 content in myotubes. ZnSO_4_ treatment significantly reduced LC3B‐II levels and restored P62 levels, indicating inhibition of excessive autophagy (Figure 4D). Immunofluorescence staining confirmed that P62 fluorescence intensity was significantly lower in HG‐treated cells compared to controls but was restored following ZnSO_4_ treatment (Supplementary Figure 2).

To further elucidate the role of autophagy in HG‐induced myotube atrophy, myotubes were treated with the autophagy agonist RAPA. In the presence of RAPA, the ZnSO_4_‐mediated reduction in LC3B‐II levels and upregulation of P62 were partially reversed (Figure 4E,F). RAPA also led to increased expression of Atrogin‐1 in myotubes. These findings suggest that ZnSO_4_ mitigates muscle atrophy by inhibiting autophagy in C2C12 myotubes.

### 3.5 ZnSO_4_ Pretreatment Activates GPR39 in Myotubes, While SIRT1 Agonist Blocks ZnSO_4_'s Anti‐Atrophic Effects

**FIGURE 5:**
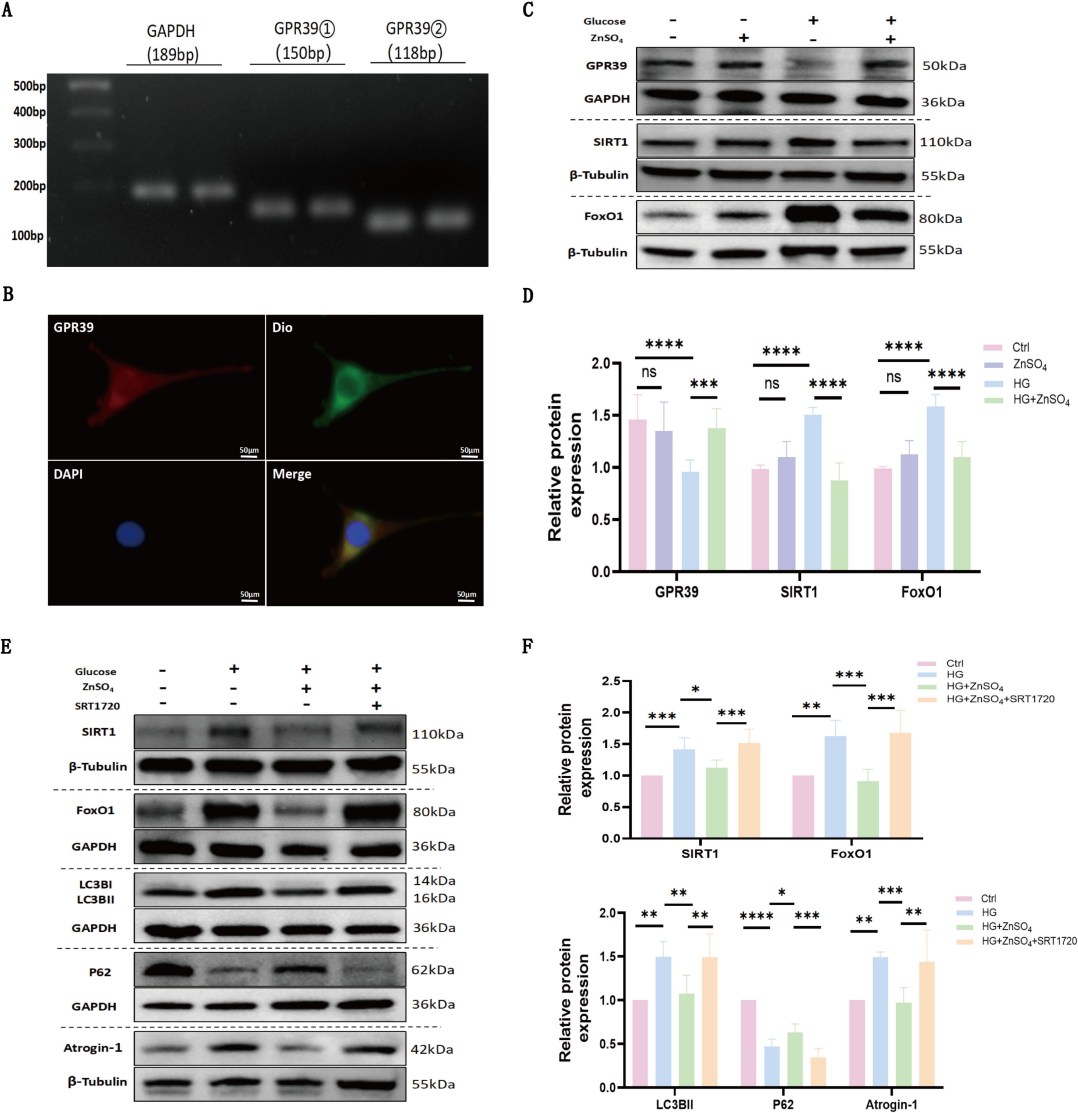
ZnSO₄ activates GPR39 in myotubes, while SIRT1 agonist blocks ZnSO₄'s anti‐atrophic effects. (A) RT‐PCR analysis of GPR39 expression in C2C12 myotubes. (B) Immunofluorescence staining of GPR39 in C2C12 myotubes. DOI is a lipophilic dye binding to cell membranes (scale bar: 50 μm; *n* = 5 independent experiments). (C–D) Western blot analysis of GPR39, SIRT1 and FoxO1 expression in fully differentiated C2C12 myotubes treated with glucose (50 mM) ± ZnSO₄ (30 μM) for 72 h. (E–F) Western blot analysis of SIRT1, FoxO1, LC3BII, P62 and Atrogin‐1 in myotubes treated with glucose (50 mM), ZnSO₄ (30 μM) and SRT1720 (1 μM) for 72 h. Data are presented as mean ± SD (*n* = 5). *p < 0.05, **p < 0.01, ***p < 0.001, ****p < 0.0001; ns: not significant.

RT‐PCR was utilized to assess GPR39 expression in C2C12 cells (Figure 5A). The internal control, GAPDH, yielded a product of 189 bp, while GPR39 mRNA was detected at 150 bp and 118 bp using two sets of primers (Figure 5A). Immunofluorescence analysis confirmed the cell membrane expression of GPR39 in the cells (Figure 5B). Western blot analysis revealed a protein band corresponding to GPR39 at approximately 50 kDa (Figure 5C). Notably, a 72‐h incubation in HG conditions resulted in decreased GPR39 expression, while significantly increasing SIRT1 and FoxO1 protein levels. Following ZnSO_4_ treatment, GPR39 protein levels were elevated, whereas SIRT1 and FoxO1 levels decreased (Figure 5C,D).

C2C12 cells were treated with ZnSO_4_ alone or in combination with the SIRT1‐specific agonist SRT1720. In cells co‐treated with ZnSO_4_ and SRT1720, SIRT1 and FoxO1 expression was significantly upregulated compared to those treated with ZnSO_4_ alone. Consistent with previous observations, ZnSO_4_ inhibited autophagy and atrophy in HG‐treated C2C12 cells. However, the protective effects of ZnSO_4_ against myotube injury in the in vitro HG model were negated by SRT1720 administration (Figure 5E,F). These findings suggest that ZnSO_4_ treatment inhibits autophagy through modulation of the SIRT1/FoxO1 pathway.

### 3.6 ZnSO_4_ Alleviates C2C12 Myotube Atrophy by Regulating SIRT1 Expression Through GPR39 to Inhibit Autophagy

**FIGURE 6:**
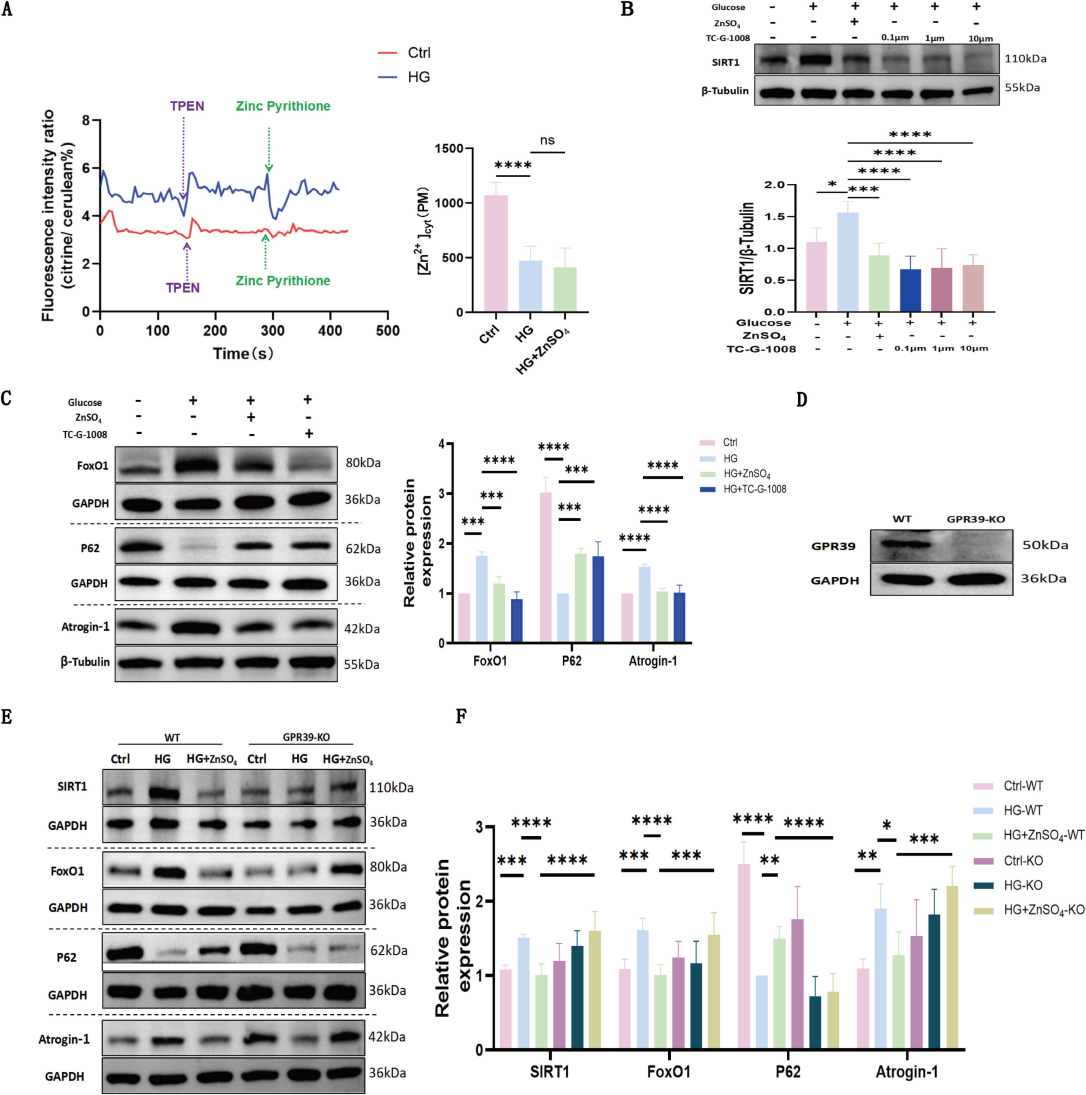
ZnSO₄ alleviates C2C12 myotube atrophy by regulating SIRT1 expression through GPR39 to inhibit autophagy. (A) Representative fluorescence traces demonstrate variations in the eCALWY‐4 fluorescence ratio, and intracellular Zn^2+^ levels were quantified from independent experiments (*n* = 6, 6, 5) by measuring the steady‐state ratio of citrine to cerulean (R) and calculating the concentration of free Zn^2+^ using the formula: [Zn^2+^] = Kd × (R_max_ − R) / (R − R_min_), where the dissociation constant (Kd) for eCALWY‐4 is 630 pM. (B)Western blot analysis of SIRT1 expression in C2C12 myotubes treated with TC‐G‐1008 (0.1, 1, 10 μM; a selective GPR39 agonist). (C) Western blot analysis of FoxO1, P62 and Atrogin‐1 expression in C2C12 myotubes treated with TC‐G‐1008 (0.1 μM). (D) CRISPR‐Cas9 knockout of GPR39 in C2C12 cells. Western blot analysis of GPR39 and GAPDH expression in wild‐type (WT) and GPR39 knockout (GPR39‐KO) cells. (E–F) Western blot analysis of SIRT1, FoxO1, P62 and Atrogin‐1 expression in WT and GPR39 KO cells treated with glucose (50 mM) ± ZnSO₄ (30 μM) for 72 h. Data are presented as mean ± SD from five independent experiments. *p < 0.05, **p < 0.01, ***p < 0.001, ****p < 0.0001. Abbreviations: KO: knockout; C‐F: *n* = 5 independent experiments; ns: not significant; WT: wild type.

FRET analysis indicated that HG treatment for 72 h reduced intracellular labile zinc concentrations. Interestingly, the addition of 30 μM Zn^2+^ to the culture medium for the same duration did not affect intracellular zinc levels (Figure 6A), suggesting that zinc may not regulate SIRT1 expression through an intracellular mechanism, a hypothesis warranting further investigation.

To explore whether GPR39 activation influences SIRT1 expression, HG‐exposed C2C12 cells were treated with the GPR39‐selective agonist TC‐G‐1008 at various concentrations, and SIRT1 expression was assessed via Western blot. Results indicated reduced SIRT1 levels in the HG+TC‐G‐1008 group compared to the HG group (Figure 6B). Similar to zinc's effects, 0.1 μM TC‐G‐1008 also decreased FoxO1 expression and mitigated HG‐induced autophagy and atrophy in C2C12 cells (Figure 6C).

The GPR39 gene was successfully knocked out using the CRISPR‐Cas9 system, establishing a GPR39‐KO cell line. Sanger sequencing revealed a 61‐base pair deletion in the GPR39 gene (Supplementary Figure 3A), and the knockout efficiency and specificity were validated by GPR39 immunofluorescence (Supplementary Figure 3B) and Western blot analysis (Figure 6D). Immunofluorescence showed near‐complete loss of GPR39 expression in KO cells compared to wild‐type (WT) cells, while Western blot confirmed the absence of GPR39 protein in KO cells. Sequence alignment using an online tool (https://ice.synthego.com/#/) reported a 100% knockout index (Supplementary Figure 3C), confirming high knockout accuracy. Atrogin‐1 levels were reduced in HG and ZnSO₄‐treated C2C12 cells but increased with GPR39 knockout (Figure 6E,F), highlighting GPR39's role in regulating C2C12 atrophy. Importantly, ZnSO₄‐induced downregulation of SIRT1 and FoxO1 was abolished by GPR39 knockout, significantly reducing ZnSO₄'s anti‐autophagic effects (Figure 6E,F). These results demonstrate that zinc regulates SIRT1 expression via GPR39 to inhibit autophagy and mitigate muscle atrophy.

## 4 Discussion

This study explored the therapeutic potential and underlying mechanisms of ZnSO₄ in alleviating STZ‐induced diabetic muscular atrophy in mice. It was revealed that zinc supplementation effectively restored serum zinc levels and attenuated muscle atrophy. Enhanced autophagy in the skeletal muscles of diabetic mice was identified through bioinformatics analyses, with FoxO1 and SIRT1 highlighted as pivotal regulatory genes. In vitro experiments further demonstrated that ZnSO₄ mitigated muscle atrophy by inhibiting the SIRT1/FoxO1 pathway and suppressing autophagy, primarily through the activation of the zinc receptor GPR39. These results offer valuable insights and a promising therapeutic strategy for combating diabetic muscular atrophy using zinc supplementation.

Zinc is an essential trace element involved in various physiological processes in the human body, including enzymatic catalysis, DNA and protein synthesis, immune regulation, and antioxidant defence. It is particularly important for proper growth and development during pregnancy, infancy, childhood, and adolescence. Additionally, zinc promotes wound healing and plays a vital role in maintaining a healthy sense of taste. In this study, the therapeutic potential of ZnSO₄ in alleviating diabetic muscular atrophy was demonstrated for the first time using an STZ‐induced diabetic mouse model. Marked hyperglycaemia was observed in diabetic mice, accompanied by significant reductions in grip strength, relative grip strength, GMI, and skeletal muscle fibre CSA, effectively replicating the pathological features of diabetic muscular atrophy. Notably, serum zinc levels were significantly reduced in T1D group; however, ZnSO_4_ treatment restored zinc homeostasis and alleviated atrophy symptoms. This correlates with clinical observations of decreased zinc levels in elderly patients with sarcopenia [23], underscoring the importance of zinc in preventing and treating this condition.

In vitro analyses further validated that ZnSO_4_ inhibited autophagy in C2C12 muscle cells exposed to HG, significantly alleviating cellular atrophy. Conversely, the use of the autophagy activator RAPA exacerbated cell atrophy, providing additional evidence for the critical role of autophagy in muscular atrophy. Apart from ZnSO_4_, other compounds have exhibited beneficial effects in addressing diabetic muscle atrophy by targeting autophagy. Specifically, Cai et al. [24] reported that niclosamide ethanolamine salt could attenuate the overexpressed autophagy‐related proteins in atrophic muscles, ultimately leading to the amelioration of muscular atrophy in T1D mice by inhibiting muscular autophagy. Similarly, Choi et al. [25] demonstrated the regulatory role of *Schisandra chinensis* extract on autophagy‐related gene expression and lysosomal function restoration, significantly mitigating muscle atrophy in STZ‐induced diabetic mice. These findings suggest that pharmacological interventions targeting excessive autophagy hold promise as strategies for treating diabetic muscle atrophy. Additionally, non‐pharmacological therapies, such as swimming exercises [26] significantly alleviated muscular atrophy symptoms in diabetic rats. The underlying mechanism of this effect might be related to the modulation of autophagy‐related gene and protein expression in muscles, thereby reducing the occurrence of excessive autophagy. Moreover, the benefits of zinc extend beyond alleviating muscle atrophy. Research [27] has indicated that zinc supplementation significantly reduces autophagy marker LC3‐II protein levels in cardiomyocytes, effectively inhibiting excessive autophagy and alleviating structural and functional abnormalities in myocardial tissues induced by a high‐fat diet and STZ in type 2 diabetic rat models. These findings not only elucidate the action mechanisms of ZnSO_4_ but also support its potential application as a therapeutic agent for diabetic muscle atrophy.

The inhibitory effect of ZnSO₄ on autophagy was investigated through the SIRT1/FoxO1 signalling pathway. SIRT1 may act both as a downstream target of ZnSO₄ and as an upstream regulator of autophagy. Recent studies have identified SIRT1 as a key modulator of autophagy [28], including its role in alleviating mitochondrial damage via the PINK1‐Parkin autophagy pathway, thereby protecting against post‐myocardial infarction remodelling in heart failure models [29]. Furthermore, SIRT1‐mediated autophagy is involved in regulating cell proliferation, metabolism, and stress resistance. The FoxO family of transcription factors is a major downstream target of SIRT1 [30]. FOXO1, in particular, is a critical transcription factor for several autophagy‐regulating genes [31]. Studies have shown that oleic and linoleic acids inhibit autophagy by downregulating the SIRT1/FoxO1 pathway, thereby promoting chondrocyte apoptosis [32]. Moreover, the SIRT1/FoxO1 axis regulates autophagy in endothelial cells, cardiomyocytes, and renal tissues [33, 34, 35]. Thus, the SIRT1/FoxO1 pathway represents a critical regulator of autophagy.

The regulatory mechanisms of SIRT1 expression in diabetic muscle atrophy are complicated. A plausible explanation is that SIRT1 upregulation may represent a compensatory response during early or specific stages of diabetic muscle atrophy. Under hyperglycaemic conditions, cells might rely on SIRT1 to enhance energy production and maintain normal physiological function. Simultaneously, SIRT1‐mediated autophagy activation may facilitate the removal of impaired organelles, protecting cells from further harm. However, it is crucial to acknowledge that excessive SIRT1 expression may lead to overactivation of autophagy, resulting in muscle protein degradation and exacerbating muscle atrophy. Our study provides preliminary evidence at the cellular level that ZnSO₄ modulates autophagy via the SIRT1/FoxO1 signalling pathway, offering new insights into potential therapeutic approaches for a range of diseases.

To elucidate the activation of SIRT1 by ZnSO₄, zinc signalling pathways mediated via both extracellular and intracellular mechanisms were explored [36]. GPR39, a zinc‐dependent G protein‐coupled receptor, was identified as a sensor of extracellular zinc, regulating zinc‐dependent signalling cascades [37]. Our results indicated that high glucose exposure reduced intracellular zinc levels, consistent with previous findings by Wang et al. [38]. This effect appears to stem from decreased expression of Slc39a5 mRNA and protein in pancreatic beta cells, correlating with reduced zinc uptake. Interestingly, when cells under HG conditions were treated with low zinc concentrations, intracellular zinc levels remained unchanged, suggesting that extracellular zinc ions may regulate SIRT1 expression through an extracellular pathway rather than direct entry into cells. Our previous research [12] demonstrated that low concentrations of zinc ions induce A20 expression via a GPR39‐dependent pathway, thereby inhibiting hypoxia‐induced proliferation and migration of pulmonary artery smooth muscle cells, showing significant therapeutic potential for pulmonary arterial hypertension. Moreover, in studies of vascular calcification, zinc was found to upregulate TNFAIP3 expression through a GPR39‐dependent pathway, ameliorating phosphate‐induced osteochondrogenic transdifferentiation and vascular calcification in vascular smooth muscle cells [39]. Overall, these findings underscore the critical role of GPR39 in mediating the protective effects of zinc.

GPR39 is widely expressed in various cell types, including vascular endothelial and vascular smooth muscle cells [39], and our research extends these observations. We detected abundant GPR39 expression in both normal muscle tissue and C2C12 cells, which was significantly reduced in STZ‐treated mice and HG‐treated C2C12 myotubes. In our study, GPR39 activation via TC‐G‐1008 was found to downregulate SIRT1 expression and inhibit HG‐induced autophagy and atrophy in C2C12 cells. Importantly, CRISPR‐Cas9‐mediated GPR39 knockout abolished zinc's inhibitory effects on the SIRT1/FoxO1 pathway, highlighting its pivotal role in this axis. The potential off‐target effects of CRISPR‐Cas9 are acknowledged. Although this study did not extensively screen for off‐target genes, multiple validation methods, including Sanger sequencing, IF, and Western blot, ensured high knockout efficiency and specificity. Online analyses further confirmed the knockout's accuracy. Future research will include whole‐genome or RNA sequencing to thoroughly evaluate off‐target effects and provide stronger evidence for GPR39's role in zinc signalling and diabetic muscle atrophy. Given zinc's other physiological roles, its protective effects against muscle atrophy likely extend beyond this pathway, potentially involving oxidative stress, inflammation, and insulin signalling. Future research will explore these interactions to uncover zinc's broader regulatory network.

This study employed a T1D model to explore the ZnSO₄'s effects on muscle atrophy. While a T2D model was not directly used, we propose zinc's therapeutic benefits are not diabetes‐type specific. Zinc has shown positive effects in both T1D and T2D by regulating glucose levels, reducing inflammation, and mitigating oxidative stress and metabolic dysfunction [Supplementary Reference S1–6]. Zinc deficiency has been shown to exacerbate diabetic complications [Supplementary Reference S7–10], while supplementation improves conditions such as nephropathy, cardiomyopathy, and insulin resistance in both forms of diabetes [Supplementary Reference S11–15]. The shared pathological mechanisms underlying muscle atrophy, including oxidative stress, chronic inflammation, and impaired autophagy, support the potential consistency of zinc's therapeutic effects in T1D and T2D, although further experimental validation is still required. We aim to include a T2D model in future studies to provide a comprehensive evaluation of zinc's therapeutic potential.

In summary, our study reported for the first time that zinc supplementation can alleviate diabetic skeletal muscle atrophy. The zinc‐sensing receptor GPR39 was found to be reduced in the gastrocnemius muscle of diabetic muscle atrophy mice. The anti‐atrophic effect of zinc on high glucose‐treated C2C12 myotubes was mediated, at least partially, through the inhibition of autophagy via the GPR39‐dependent SIRT1/FoxO1 signalling pathway. These findings provide a foundation for further exploration of zinc supplementation as a potential therapeutic strategy for muscle atrophy in diabetes.

## Conflicts of Interest

The authors declare no conflicts of interest.

## Supporting information


**Supplementary Fig 1** Bioinformatics analysis of DEGs. (A) Heatmap of autophagy‐related upregulated DEGs in T1D atrophic muscles. (B) Gene set enrichment analysis (GSEA) for the ‘autophagy’ pathway. (C) RT‐qPCR analysis of BCL2L11 and GSK3β expression in GM.

**Supplementary Fig 2** Immunofluorescence staining of P62 in C2C12 myotubes treated with glucose ± ZnSO₄ (30 μM) for 72 h

**Supplementary Fig 3** Validation of GPR39 knockout in CRISPR‐Cas9‐treated cell lines. (A) Sanger sequencing of the target genomic DNA. (B) Immunofluorescence staining of GPR39. (C) Sequence alignment using an online analysis tool.

**Supplementary Table 1** Sequence of target mRNA
Supplementary Table 2. Top 10 hub genes obtained by six algorithms of cytoHubba
