# Supplementary figures and images for: Zinc Alleviates Diabetic Muscle Atrophy via Modulation of the SIRT1/FoxO1 Autophagy Pathway Through GPR39
Source: J Cachexia Sarcopenia Muscle. 2025 Mar 3;16(2):e13771. doi: 10.1002/jcsm.13771 (PMC11873538; doi:10.1002/jcsm.13771)

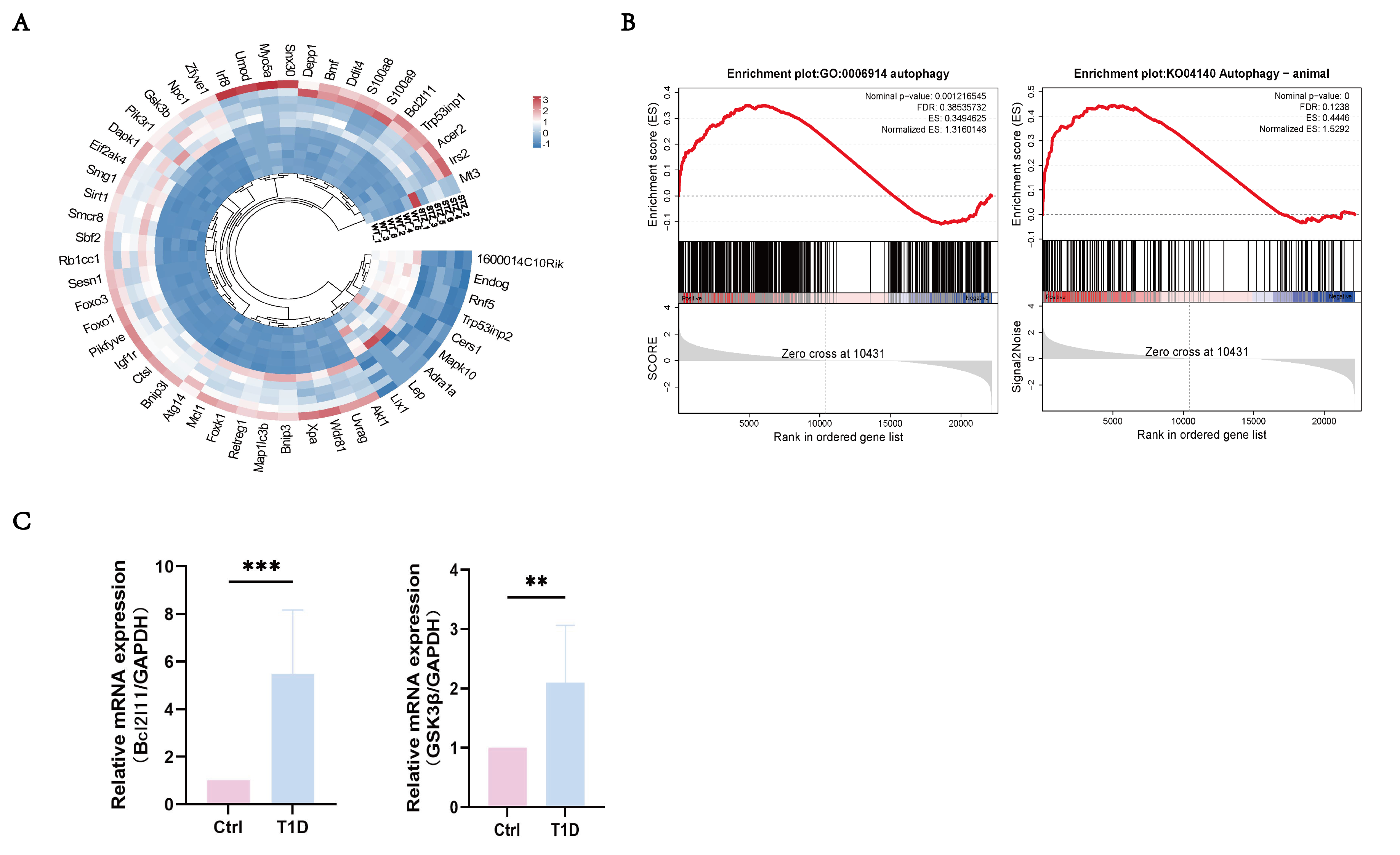

Supplement: Supplementary file 1 — Supplementary Fig 1 Bioinformatics analysis of DEGs. (A) Heatmap of autophagy‐related upregulated DEGs in T1D atrophic muscles. (B) Gene set enrichment analysis (GSEA) for the ‘autophagy’ pathway. (C) RT‐qPCR analysis of BCL2L11 and GSK3β expression in GM. [file JCSM-16-e13771-s003.tif]

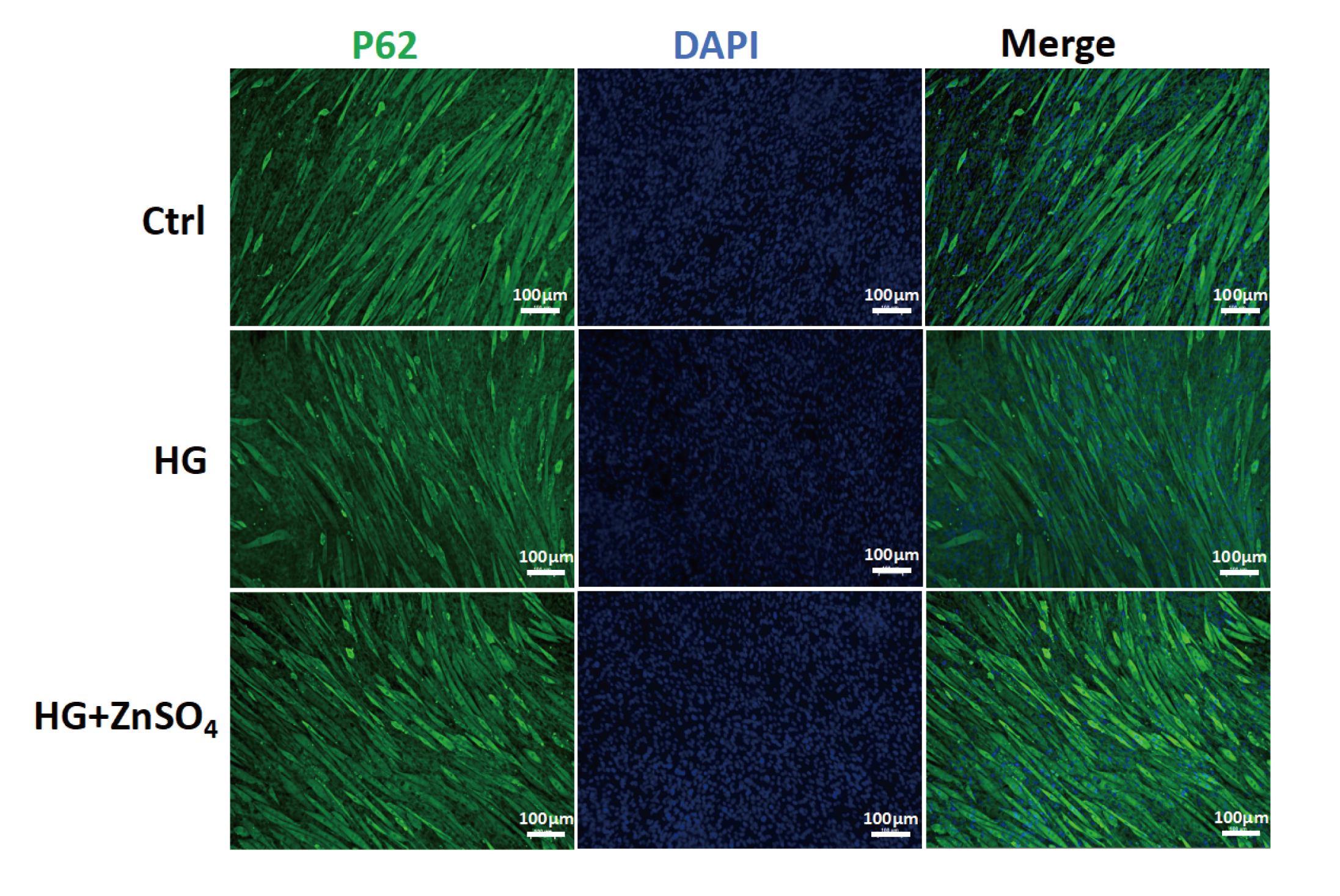

Supplement: Supplementary file 2 — Supplementary Fig 2 Immunofluorescence staining of P62 in C2C12 myotubes treated with glucose ± ZnSO₄ (30 μM) for 72 h [file JCSM-16-e13771-s004.tif]

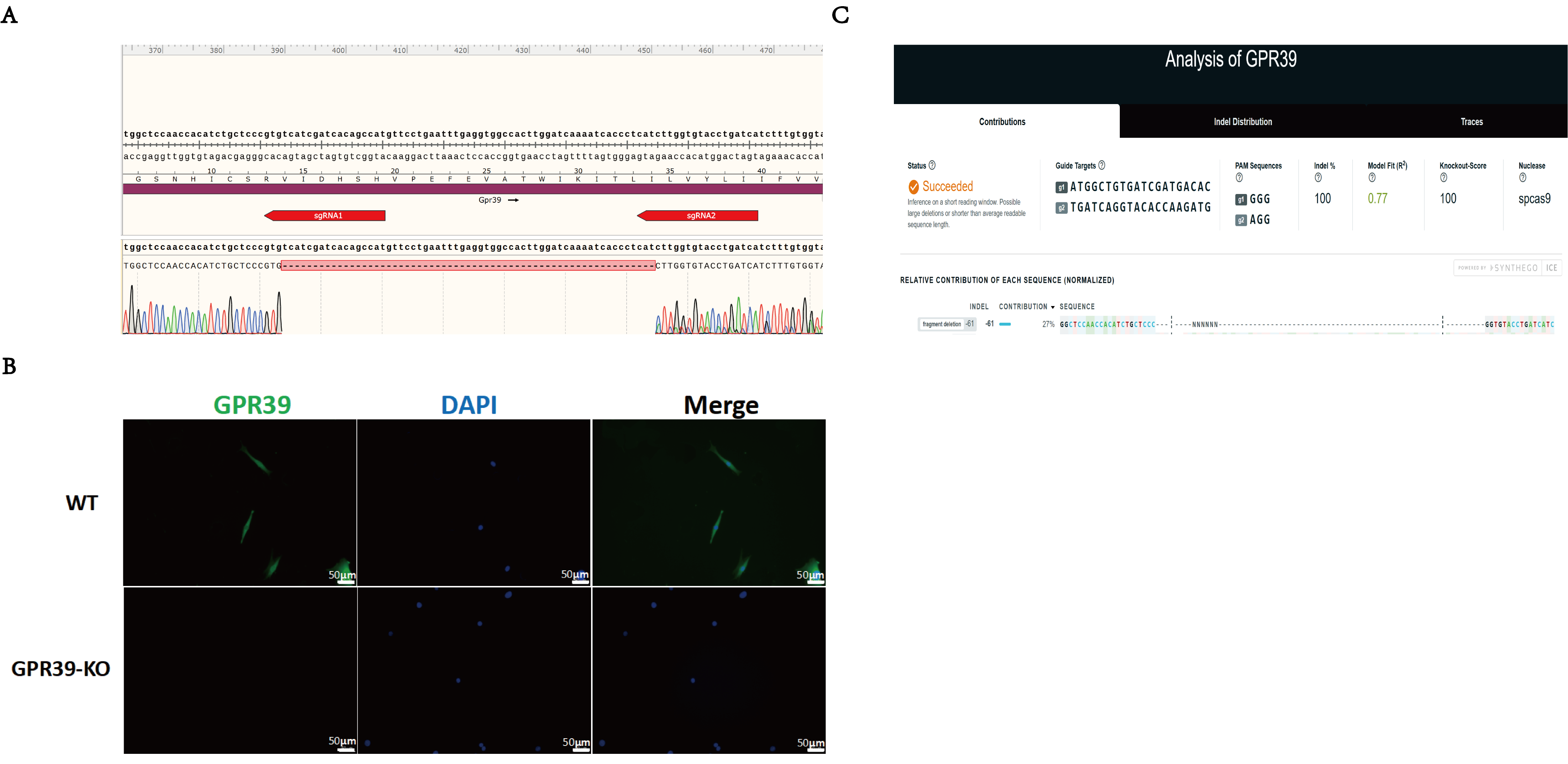

Supplement: Supplementary file 3 — Supplementary Fig 3 Validation of GPR39 knockout in CRISPR‐Cas9‐treated cell lines. (A) Sanger sequencing of the target genomic DNA. (B) Immunofluorescence staining of GPR39. (C) Sequence alignment using an online analysis tool. [file JCSM-16-e13771-s002.tif]
