# Supplementary material for: Zinc Alleviates Diabetic Muscle Atrophy via Modulation of the SIRT1/FoxO1 Autophagy Pathway Through GPR39
Source: J Cachexia Sarcopenia Muscle. 2025 Mar 3;16(2):e13771. doi: 10.1002/jcsm.13771 (PMC11873538; doi:10.1002/jcsm.13771)
Supplement: Supplementary file 4 — Supplementary Table 1 Sequence of target mRNA Supplementary Table 2. Top 10 hub genes obtained by six algorithms of cytoHubba [file JCSM-16-e13771-s001.docx]

**Supporting Information**

**Zinc Alleviates Diabetic Muscle Atrophy via Modulation of the SIRT1/FoxO1 Autophagy Pathway through GPR39**

Xing Yu ^1,2,3,4,5†^,Xiaojun Chen ^1,2,3,4,5†^ ,Weibin Wu^1,2,3,4,5†^ ,Huibin Tang ^1,2,3,4,5^,Yunyun Su ^1,2,3,4,5^,Guili Lian^1,2,3,4,5^,Yujie Zhang^1,2*^,Liangdi Xie ^1,2,3,4,5*^

# **Supplementary Methods**

**1.1 Histological analysis**

Sections were stained with hematoxylin and eosin (HE)for morphological evaluation, and the cross-sectional area (CSA) of muscle fibers was analyzed using ImageJ software (NIH, Bethesda, MD, USA).

For immunofluorescence (IF) staining, the protocol utilized in our prior research was followed[19]. The primary antibodies utilized included:antibodies against Atrogin-1 (1:200, Cat. No. ab168372, Abcam, USA), LC3B (1:200, Cat. No. 83506, Cell Signaling Technology, USA), P62 (1:200, Cat. No. A19700, ABclonal, China), and GPR39 (1:50, Cat. No. AGR-045, Alomone, Israel).

For immunohistochemical(IHC) staining, the protocol utilized in our prior research was followed[19].The primary antibodies utilized included:antibodies againstSIRT1 (1:500, Cat. No. ab189494, Abcam, USA), FoxO1 (1:200, Cat. No. 2880, Cell Signaling Technology, USA), and GPR39 (1:100, Cat. No. NLS139, NOVUS, USA).

**1.2 Transmission electron microscope (TEM)**

The detailed protocol for TEM of muscle tissues has been previously described[19]. Briefly, tissue samples were cut into 1 mm³ cubes and fixed with 2.5% glutaraldehyde. Both tissue and cell samples were embedded in resin for ultrathin sectioning. Sections were stained with uranyl acetate and lead citrate, and images were captured using a transmission electron microscope (HT7800, HITACHI, China).

**1.3 Western blot**

Western blot analysis was conducted as previously described[19]. The primary antibodies utilized included: Atrogin-1 (1:1000, Cat. No. ab168372, Abcam, USA), MuRF1 (1:1000, Cat. No. 55456-1-AP, Proteintech, China), LC3B (1:1000, Cat. No. 83506, Cell Signaling Technology, USA), P62 (1:1000, Cat. No. A19700, ABclonal, China), SIRT1 (1:1000, Cat. No. ab189494, Abcam, USA), FoxO1 (1:1000, Cat. No. 2880, Cell Signaling Technology, USA), GPR39 (1:1000, Cat. No. NLS139, NOVUS, USA), and GAPDH (1:1000, Cat. No. 10494-1-AP, Proteintech, China).

**1.4 Cell IF staining**

Myotube cells were fixed in 4% paraformaldehyde for 15 minutes at room temperature, permeabilized with 0.25% Triton X-100 for 10 minutes at 4℃, and blocked with 5% BSA for 30 minutes at room temperature. Cells were incubated overnight at 4℃ with anti-MHC1 antibody (1:100, Cat No. MF 20, DSHB, USA) and anti-P62 antibody (1:200, Cat. No. A19700, ABclonal, China). Following primary antibody incubation, cells were washed with PBS and incubated with Alexa Fluor 488-labeled secondary antibodies (1:200) for 1 hour, followed by DAPI counterstaining.

To assess the cellular localization of GPR39, C2C12 myoblasts were treated with the green fluorescent cell membrane probe DiO (Cat. No: M5121, ABMole, China) for 10 minutes before staining. Subsequently, the remaining steps were carried out as described above, with cells incubated overnight with anti-GPR39 antibody (1:50, Cat. No. AGR-045, Alomone, Israel).

# **2.Supplementary tables**

**Supplementary Table 1. Sequence of target mRNA**

| Target RNA | Primers | Sequence |
| --- | --- | --- |
| MuRF-1 | F | 5'-CAGCTGATCTGCCCCATCTG-3' |
|  | R | 5'-AACGGAAACGACCTCCAGAC-3' |
| Atrogin-1 | F | 5'-GGACTTCTCGACTGCCATCC-3' |
|  | R | 5'-AGAGTCTGGAGAAGTTCCCGT-3' |
| GAPDH | F | 5'-TGGAAAGCTGTGGCGTGATG-3' |
|  | R | 5'-TGGAAAGCTGTGGCGTGATG-3' |
| GSK3β | F | 5'-CCTCCACATGCTCGGATTCA-3' |
|  | R | 5'-CAGAAGCGGCGTTATTGGTC-3' |
| SIRT1 | F | 5'-ACCACCAAATCGTTACATATTCCA-3' |
|  | R | 5'-CCGTATCATCTTCCAAGCCATT-3' |
| FOXO1 | F | 5'-ATTCGGTCATGCCAGCGTAT-3' |
|  | R | 5'-CAAGCGGTTCATGGCAGATG-3' |
| Bcl2l11 | F | 5'-TGGCCAAGCAACCTTCTGAT-3' |
|  | R | 5'-GCGGTTCTGTCTGTAGGGAG-3' |
| GPR39 | F | 5'-GCTACATTGCCATTTGTCATCC-3' |
|  | R | 5'-CGTTTACCAGAGGGTACTCGA-3' |
| GPR39 | F | 5'-CATGTTCCTGAATTTGAGGTGGC-3' |
|  | R | 5'-ATACCTGCGTAACCCTGATGG-3' |

**Supplementary Table 2. Top ten hub genes obtained by six algorithms of cytoHubba**

| **MCC** | **DNNC** | **MNC** | **DEGREE** | **EPC** | **Radiality** |
| --- | --- | --- | --- | --- | --- |
| Akt1 | Mapk10 | Akt1 | Akt1 | Akt1 | Akt1 |
| Foxo3 | Igf1r | Foxo3 | Foxo3 | Foxo3 | Bnip3 |
| Gsk3b | Lep | Bnip3 | Map1lc3b | Map1lc3b | Foxo3 |
| Igf1r | Irs2 | Map1lc3b | Bnip3 | Bnip3 | Map1lc3b |
| Mapk10 | Foxo1 | Gsk3b | Sirt1 | Gsk3b | Sirt1 |
| Sirt1 | Bcl2l11 | Sirt1 | Gsk3b | Sirt1 | Uvrag |
| Foxo1 | Gsk3b | Foxo1 | Foxo1 | Foxo1 | Bcl2l11 |
| Bcl2l11 | Mcl1 | Bcl2l11 | Bcl2l11 | Mcl1 | Rb1cc1 |
| Irs2 | Sirt1 | Mcl1 | Mcl1 | Bcl2l11 | Foxo1 |
| Mcl1 | Atg14 | Uvrag | Rb1cc1 | Igf1r | Gsk3b |

# **3.Supplementary references**

[1] Simon SF, Taylor CG. Dietary zinc supplementation attenuates hyperglycemia in db/db mice. Exp Biol Med (Maywood). 2001;226(1):43–51.

[2] Faure P, Benhamou PY, Perard A, Halimi S, Roussel AM. Lipid peroxidation in insulin-dependent diabetic patients with early retina degenerative lesions: effects of an oral zinc supplementation. Eur J Clin Nutr. 1995;49(4):282–8.

[3] Shidfar F, Aghasi M, Vafa M, Heydari I, Hosseini S, Shidfar S. Effects of combination of zinc and vitamin A supplementation on serum fasting blood sugar, insulin, apoprotein B and apoprotein A-I in patients with type i diabetes. Int J Food Sci Nutr. 2010;61(2):182–91.

[4] Afkhami-Ardekani M, Karimi M, Mohammadi SM, Nourani F. Effect of zinc sulfate supplementation on lipid and glucose in type 2 diabetic patients.Pak J Nutr. 2008;7(4):550–3.

[5] Al-Maroof RA, Al-Sharbatti SS. Serum zinc levels in diabetic patients and effect of zinc supplementation on glycemic control of type 2 diabetics. Saudi Med J. 2006;27(3):344–50.

[6] Jayawardena R, Ranasinghe P, Galappatthy P, Malkanthi R, Constantine G, Katulanda P. Effects of zinc supplementation on diabetes mellitus: a systematic review and meta-analysis. Diabetol Metab Syndr. 2012;4(1):13.

[7]Fushimi H, Inoue T, Yamada Y, Horie H, Kameyama M, Inoue K, et al. Zinc deficiency exaggerates diabetic osteoporosis. Diabetes Res. Clin. Pr. 1993, 20, 191–196.

[8]Zhang C,Lu X,Tan Y, Li B,Miao X,Jin L,et al. Diabetes-induced hepatic pathogenic damage, inflammation, oxidative stress, and insulin resistance was exacerbated in zinc deficient mouse model. PLoS ONE 2012, 7, e49257.

[9]Miao X,Sun WX, Miao LN,Fu YW,Wang YG,Su GF,et al. Zinc and diabetic retinopathy. J. Diabetes Res. 2013, 425854.

[10]Milnerowicz H,Jablonowska M, Bizon A. Change of zinc, copper, and metallothionein concentrations and the copper-zinc superoxide dismutase activity in patients with pancreatitis. Pancreas 2009, 38, 681–688.

[11]Gembillo G, [Visconti](https://pubmed.ncbi.nlm.nih.gov/?sort=date&term=Visconti+L&cauthor_id=35405968)  L,  [Giuffrida](https://pubmed.ncbi.nlm.nih.gov/?sort=date&term=Giuffrida+AE&cauthor_id=35405968) AE,  [Labbozzetta](https://pubmed.ncbi.nlm.nih.gov/?sort=date&term=Labbozzetta+V&cauthor_id=35405968) V, [Peritore](https://pubmed.ncbi.nlm.nih.gov/?sort=date&term=Peritore+L&cauthor_id=35405968) L, [Lipari](https://pubmed.ncbi.nlm.nih.gov/?sort=date&term=Lipari+A&cauthor_id=35405968) A, et al. Role of Zinc in Diabetic Kidney Disease. Nutrients, 2022, 14(7): 1353.

[12]Özcelik D, [Nazıroglu](https://pubmed.ncbi.nlm.nih.gov/?sort=date&term=Naz%C4%B1roglu+M&cauthor_id=23054862) M, [Tunçdemir](https://pubmed.ncbi.nlm.nih.gov/?sort=date&term=Tun%C3%A7demir+M&cauthor_id=23054862) M, [Çelik](https://pubmed.ncbi.nlm.nih.gov/?sort=date&term=%C3%87elik+%C3%96&cauthor_id=23054862) Ö, [Öztürk](https://pubmed.ncbi.nlm.nih.gov/?sort=date&term=%C3%96zt%C3%BCrk+M&cauthor_id=23054862) M, [Flores-Arce](https://pubmed.ncbi.nlm.nih.gov/?sort=date&term=Flores-Arce+MF&cauthor_id=23054862) MF. Zinc Supplementation Attenuates Metallothionein and Oxidative Stress Changes in Kidney of Streptozotocin-Induced Diabetic Rats. Biological Trace Element Research, 2012, 150(1-3): 342-349.

[13][Yang](https://pubmed.ncbi.nlm.nih.gov/?sort=date&term=Yang+F&cauthor_id=28595780) F, [Li](https://pubmed.ncbi.nlm.nih.gov/?sort=date&term=Li+B&cauthor_id=28595780) B,  [Dong](https://pubmed.ncbi.nlm.nih.gov/?sort=date&term=Dong+X&cauthor_id=28595780) XM, [Cui](https://pubmed.ncbi.nlm.nih.gov/?sort=date&term=Cui+W&cauthor_id=28595780) WP, [Luo](https://pubmed.ncbi.nlm.nih.gov/?sort=date&term=Luo+P&cauthor_id=28595780) P. The Beneficial Effects of Zinc on Diabetes-induced Kidney Damage in Murine Rodent Model of Type 1 Diabetes Mellitus.J Trace Elem Med Biol.2017:42:1-10.

[14] Wang SY, Nie P, Lu XD, Li CG, Dong XM, Yang F, et al. Nrf2 Participates in the Anti-apoptotic Role of Zinc in Type 2 Diabetic Nephropathy through Wnt/β-catenin Signaling Pathway. Journal of Nutritional Biochemistry.2020 :84:108451.

[15]Cai L, Tan Y, Watson S, Wintergerst K. Diabetic Cardiomyopathy–Zinc Preventive and Therapeutic Potentials by Its Anti-oxidative Stress and Sensitizing Insulin Signaling Pathways.Toxicol Appl Pharmacol.2023 :477:116694.
